# Editorial: Therapeutics in pulmonary arterial hypertension

**DOI:** 10.3389/fcvm.2024.1463305

**Published:** 2024-08-06

**Authors:** Rui Adão, Francisco Perez-Vizcaino, Bassam Redwan, Carmen Brás-Silva

**Affiliations:** ^1^ Cardiovascular R&D Centre-UnIC@RISE, Department of Surgery and Physiology, Faculty of Medicine, University of Porto, Porto, Portugal; ^2^ Department of Pharmacology and Toxicology, School of Medicine, University Complutense of Madrid, Madrid, Spain; ^3^ CIBER Enfermedades Respiratorias (Ciberes), Madrid, Spain; ^4^ Instituto de Investigación Sanitaria Gregorio Marañón (IiSGM), Madrid, Spain; ^5^ Department of Thoracic Surgery, Klinik am Park, Klinikum Westfalen, Lünen, Germany; ^6^ Faculty of Medicine, University of Witten Herdecke, Witten, Germany; ^7^ Faculty of Nutrition and Food Sciences, University of Porto, Porto, Portugal

**Keywords:** pulmonary hypertension, right ventricle, vascular diseases, right heart failure, therapy, endothelial dysfunction, pulmonary vascular remodeling

**Editorial on the Research Topic**
Therapeutics in pulmonary arterial hypertension

Pulmonary arterial hypertension (PAH) is characterized by significant morbidity and mortality and despite advances, the prognosis remains poor, highlighting the critical need for new therapies and non-invasive methods to monitor disease progression. Although defined by strict hemodynamic criteria, PAH is a syndrome based on diverse etiologies and pathogenesis (1, 2). This Research Topic aimed to highlight the recent advances in PAH research and includes 9 papers, comprising 4 original research articles (basic and translational), 4 reviews and 1 opinion article.

In this issue, 2 outstanding basic research original studies were published. Recent investigations demonstrated that the thromboxane (TX) A2 receptor (TP) antagonist NTP42 attenuates experimental PAH across key hemodynamic parameters in the lungs and heart (3), and Mulvaney et al. aimed to validate the efficacy of *NTP42:KVA4*, a novel oral formulation of NTP42 recently validated in a Phase I clinical trial (4), in experimental PAH. Together, the findings from two independent preclinical models (monocrotaline and pulmonary artery banding) demonstrated that *NTP42:KVA4* not only alleviates pulmonary pathologies akin to those observed in clinical PAH, but also may act as a direct cardioprotective agent in settings of right ventricle (RV) pressure overload. This is relevant because RV function is widely viewed as the most important determinant of clinical outcome in PAH (5). This work points to a cardioprotective effect for *NTP42:KVA4* as a component of its potential to be a disease-modifying therapy in PAH and other cardiac conditions.

In the last two decades, more than 20 genes have been linked to a genetic predisposition to PAH, including those encoding ATP-sensitive K + channels (KATP) (6). The ABCC9 gene encodes two regulatory subunits of KATP channels: SUR2A and SUR2B. Le Ribeuz et al. showed that Kir6.1 and SUR2 are expressed in human and rat lungs and in pulmonary artery smooth muscle cells (PASMC) and PA endothelial cells (PAEC). SUR2A is upregulated in PASMCs from PAH patients but downregulated in rat lungs with monocrotaline-induced PAH. The KATP channel opener pinacidil inhibited proliferation in healthy but not PAH endothelial cells and reduced the proliferation and migration of control and PAH-PASMCs. Pinacidil caused stronger relaxation in human control compared to PAH pulmonary arteries, a result not replicated in the rat model, and preventive or curative treatment with pinacidil in vivo reduced the severity of experimental PAH. These findings suggest complex changes in KATP channels in PAH and indicate that the monocrotaline model may not accurately reflect human PAH. However, SUR2 activators might effectively treat human PAH through vasodilator and antiproliferative effects on PASMC. Further studies are needed to confirm these interesting findings and explore therapeutic benefits.

In this issue, 4 comprehensive reviews were published. Mamazhakypov and Lother discuss the recent advances in mineralocorticoid receptor (MR) signaling in pulmonary vascular cells based on preclinical research and the potential in bringing MR antagonists (MRAs) into clinical application. During the past decade, a series of experimental studies investigated the role of aldosterone and its receptor, the MR, in pulmonary vascular remodeling and a potential benefit of MRAs for PH patients. Indeed, MR is an important and highly versatile transcription factor that regulates various key signaling pathways in the PH pathogenesis, inducing adverse cellular processes that lead to pulmonary vascular remodeling, such as endothelial cell apoptosis, smooth muscle cell proliferation, pulmonary vascular fibrosis, and inflammation (7). In other very interesting review, Santos-Gomes et al. revise potential circulating biomarkers for PAH, aiming to enhance diagnostic accuracy and therapeutic monitoring. This review underscores the evolving landscape of PAH management through innovative biomarker-driven approaches. In fact, RV catheterization is the current gold standard for diagnosis (1), but its invasiveness limits routine use, and, in this context, biomarkers show promise in predicting prognosis and therapy response if they are easy to detect and objective. Also, in this issue, Simmons Beck et al. review two transcription factors, SRY-box transcription factor 17 (SOX17) and one of its downstream targets, Runt-related transcription factor 1 (RUNX1), and the emerging data that implicate their roles in the pathogenesis of PAH, from their own work and other studies. Preclinical studies in endothelial cell SOX17 deficient mice or transgenic mice with mutations SOX17 resembling those present in some PAH patients have replicated many of the pathologic features of human PAH. Consistently, SOX17 downstream targets such as Notch signaling or BMPR2 have been widely involved in PAH. Also, RUNX1 may play a role in mediating the effect of impaired SOX17 expression in PAH and targeted deletion of RUNX1 in either myeloid or endothelial cells or pharmacological inhibition of RUNX1 reverse experimental PAH. Thus, they propose that RUNX1 inhibition may be an effective approach to treat PAH. Also, Körbelin et al. comprehensively review the pivotal role of dysregulated transcription factors (TFs) in PAH pathogenesis and underscore their potential as targets for vasculoregenerative or reverse remodelling therapies. Indeed, PAH arises from dysfunction in vessel wall cells and remodelling of the pulmonary vasculature, and TFs, pivotal in regulating gene expression, intricately modulate these responses through complex networks. Complex TF networks and chromatin remodeling add layers of complexity. While emerging TF-based therapies hold promise, addressing off-target effects, possibly through gene therapy (8), is crucial. Moreover, validation through clinical trials is imperative for advancing these innovative treatments effectively.

In the landscape of clinical hypertension research, 3 published articles have provided crucial insights into the management and prognosis these patients. Over the years, PAH-specific therapies have significantly enhanced patient survival. One such therapy is selexipag, an oral selective prostacyclin receptor agonist approved for PAH treatment (1). In the study from Ciu et al., use of selexipag in triple combination therapy has shown promise in improving outcomes for Chinese PAH patients. While selexipag has demonstrated efficacy in reducing clinical worsening events and improving right heart parameters, further research is needed to fully understand its benefits in specific patient populations like the Chinese. The evolving landscape of PAH treatment strategies underscores the importance of continued investigation into optimizing therapies to enhance patient outcomes.

In terms of assessment and management PAH represents a clinical challenge and despite advancements in risk stratification and specialized care, individual patients with PAH remain difficult to evaluate accurately (9). Thus, the use of linear models to evaluate pulmonary and systemic pressure in PAH patients as described by Doyle et al. offers a promising approach to address existing paradoxes in understanding these conditions. These models provide a comprehensive set of metrics that can aid in monitoring patients without the need for invasive procedures like right heart catheterization. By directly assessing pressure and cardiac status at a component level, these models have the potential to facilitate the translation of therapeutic benefits between different trials.

In recent years, the widespread use of mobile phones has raised concerns about their potential impact on health, particularly in relation to hypertension (10) and, in an opinion article, Bhattacharya et al. shed light on the association between mobile phone usage and the development of hypertension. The study highlighted that mobile phone users had a 7% increased risk of developing hypertension compared to non-users over a 12-year follow-up period, emphasizing the need to consider mobile phone guidelines in hypertension management protocols.

All these new findings and updated reviews collectively contribute to advancing our understanding of PAH pathophysiology and managing and treatment strategies, paving the way for improved care and outcomes for patients with this debilitating condition. However, ever more research is crucial to explore existing and innovative therapies that could further improve patient prognosis and survival.
